# A case of esophagogastric junction obstruction due to octopus ingestion

**DOI:** 10.1002/jgf2.765

**Published:** 2024-12-29

**Authors:** Junya Shimamoto, Hironori Nakahira

**Affiliations:** ^1^ Department of General Medicine Kokuho Ipponmatsu Hospital Minami Uwa‐gun Japan; ^2^ Department of General Medicine Fellow of Ehime Prefectural Minamiuwa Hospital Ehime Japan

**Keywords:** aspiration risk, diagnostic imaging, esophageal obstruction, octopus ingestion, push technique

## Abstract

Endoscopic image of the stomach. A mass of octopus is obstructing the entrance to the esophagogastric junction.

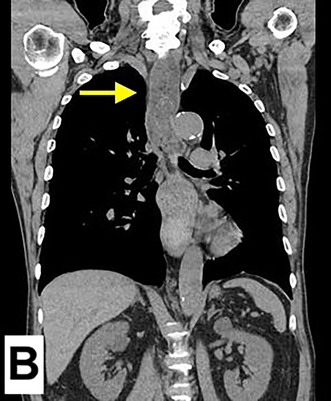

A 77‐year‐old man presented to the emergency department with chest constriction and persistent vomiting after consuming chopped octopus. A non‐contrast CT scan revealed esophageal dilation with a hyperdense mass obstructing the esophagogastric junction (Figure 1A) and food residues extending into the cervical esophagus (Figure 1B), suggesting significant obstruction and increasing the risk of aspiration. To reduce this risk and improve visibility during endoscopy, a nasogastric tube was inserted approximately 30 cm through the nasal passage, and approximately 50 mL of yellowish, muddy food residues were aspirated. Subsequent esophagogastroduodenoscopy identified an octopus lodged in the distal esophagus (Figure 2). Attempts to remove the octopus using forceps or a retrieval basket were unsuccessful due to its slippery texture, which caused it to disintegrate. Consequently, the push technique^1^ was employed, successfully advancing the octopus into the stomach and confirming its passage through the esophagogastric junction. Post‐procedural evaluation confirmed that both the stomach and esophagus were intact.

**FIGURE 1:**
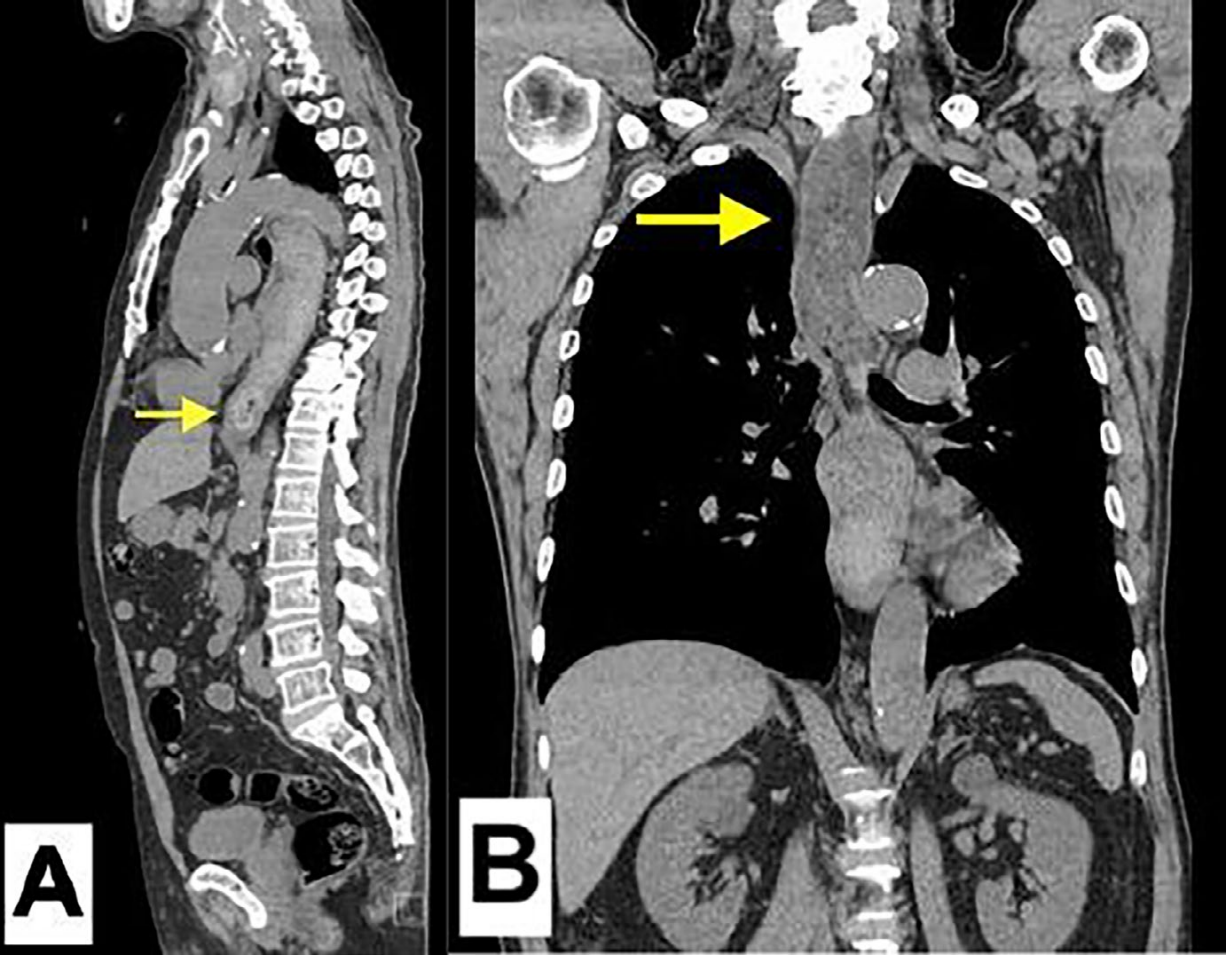
CT images of esophageal obstruction caused by octopus ingestion. (A) Axial CT scan shows an obstruction at the esophagogastric junction. The yellow arrow identifies an octopus fragment lodged at the esophagogastric junction, resulting in partial obstruction. (B) Coronal CT scan shows retained food residues, as indicated by the yellow arrow, caused by an obstruction at the esophagogastric junction and extending proximally to the cervical esophagus.

**FIGURE 2:**
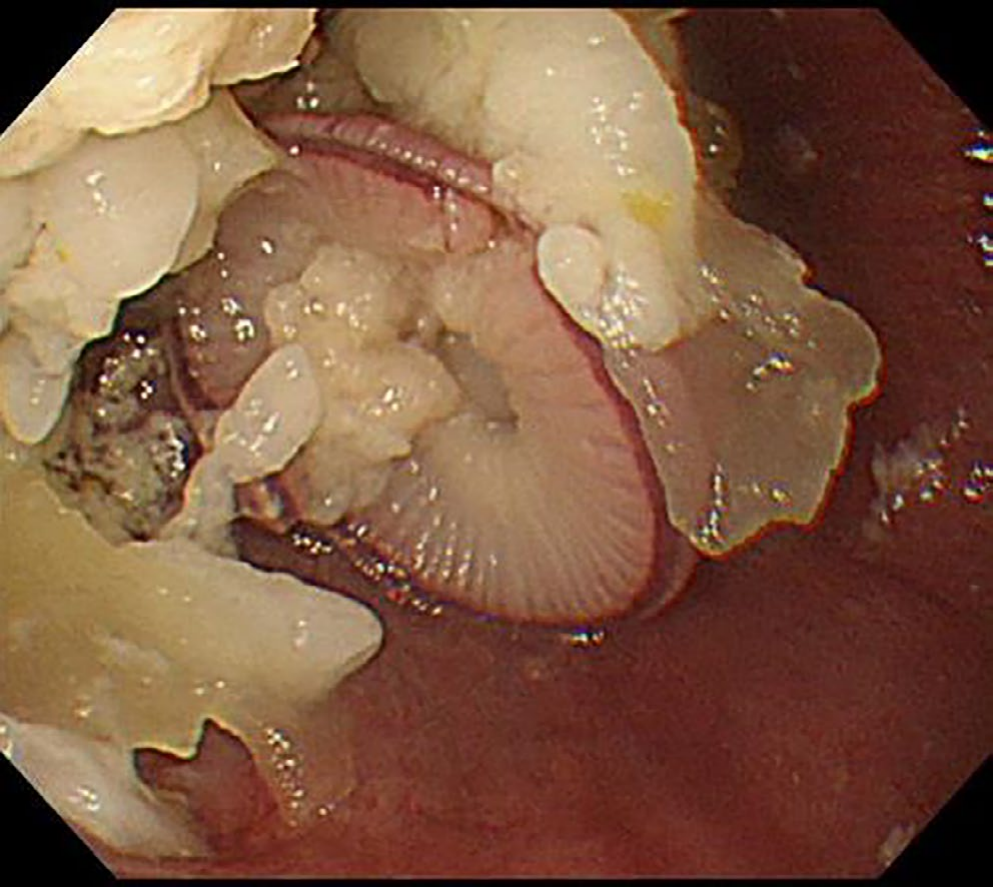
Endoscopic image of the stomach. A mass of octopus is obstructing the entrance to the esophagogastric junction. Multiple device‐assisted removal attempts were unsuccessful. Ultimately, the octopus was pushed into the stomach using the push technique.

This clinical image report emphasizes two key points. First, it highlights the importance of imaging in the management of patients with gastrointestinal obstruction symptoms. Clinicians must recognize that esophageal obstructions pose a particularly high risk of asphyxiation due to their proximity to the airway, making thorough evaluation and careful preparation essential. While fatalities due to aspiration have been reported even in cases of distal gastrointestinal obstruction under general anesthesia,^2^ esophageal obstructions require even greater caution. This case underscores the dangers of attempting upper endoscopy without adequate precautions and demonstrates the necessity of assessing aspiration risks beforehand.

Second, this report focuses on the rarity of esophageal obstruction caused by octopus ingestion. Such cases are exceedingly rare, and reports featuring detailed imaging are even less common. Unlike previous reports, which primarily focused on removal techniques, this case emphasizes the diagnostic and procedural value of pre‐endoscopic CT and endoscopic imaging. By placing greater emphasis on imaging, this report offers valuable insights into the safe and effective management of such unique and challenging cases.

## AUTHOR CONTRIBUTIONS

JS and HN conducted the endoscopic removal of Octopus. JS wrote the first draft and managed the submission process, and HN checked the draft.

## FUNDING INFORMATION

None.

## CONFLICT OF INTEREST STATEMENT

The authors declare no conflict of interest.

## PATIENT CONSENT

Written informed consent for publication and confidentiality was obtained from the patient.

## ETHICS STATEMENT

We confirm that written informed consent was obtained from the patient for publication of the clinical images and accompanying text. The patient was informed that the images and text would be published in a journal accessible to the public, and anonymity was ensured by omitting identifiable information.
